# Effectiveness and safety of imeglimin in routine clinical practice among Indian patients with type 2 diabetes mellitus: a prospective, non-interventional, real-world observational study (INDI-TIMES 2.0)

**DOI:** 10.3389/fendo.2026.1973270

**Published:** 2026-10-12

**Authors:** Debmalya Sanyal, Anuj Maheshwari, Subrata Chakrabarti, Manoj Chawla, Mangesh Tiwaskar, Soujanya Kunapa Reddy, S Sridhar, Arthur Joseph Ashirvatham, Rituraj Benode Deb, Gayathry V M

**Affiliations:** 1 School Of Medicine and Public Health, The Newcastle University, Newcastle, NSW, Australia; 2 Narayana Health Rabindranath Tagore International Institute Of Cardiac Sciences, Eastern Metropolitan Bypass, Mukundapur Market, Stadium Colony, Mukundapur, Kolkata, West Bengal, India; 3 Kali Prasad Chowdhury Medical College and Hospital, Jadavpur, Kolkata, West Bengal, India; 4 Department of Medicine, Hind Institute of Medical Sciences, Sitapur, India; 5 Binapani Doctors Clinic, Khelar Mat, University, West Bengal, Kolkata, India; 6 Lina Diabetes Care Mumbai Diabetes Research Center, Cosmos Plaza, Opp. Indian Oil Nagar, Andheri West, Mumbai, India; 7 Dept. of Medicine, Shilpa Medical Research Center, Awadhoot Nagar, Dahisar East, Mumbai, Maharashtra, India; 8 ReLiv Healthy India Pvt.Ltd., MLA Colony, Banjara Hills, Hyderabad, Telangana, India; 9 Zyva Life, Ambedkar Colony, Film Nagar, Hyderabad, Telangana, India; 10 Miot Hospital, Chennai, Sivasami Salai, Mylapore, Chennai, India; 11 Chokkikulam, Tamil Nadu, Madurai, India; 12 Zydus Healthcare Limited, Zydus Tower, Goregaon (E), Mumbai, India

**Keywords:** glimins, HbA1c, imeglimin, indi-times 2.0, real-world study, type 2 diabetes mellitus

## Abstract

**Introduction:**

Imeglimin is the first agent of the ‘*glimin’* class of glucose-lowering medication. Clinical trials have demonstrated that imeglimin is effective and well-tolerated in patients with Type 2 Diabetes Mellitus (T2DM). This prospective, non-interventional, single-arm, open-label, multi-center observational study evaluates the effectiveness and safety of imeglimin in routine clinical practice in India.

**Methods:**

The study was conducted across 145 centers in India. Adults (≥18 years) with T2DM who were prescribed imeglimin 1000 mg twice daily, either as monotherapy or as add-on therapy, were enrolled and followed for 16 weeks. The primary endpoint was the change in glycated hemoglobin (HbA1c) from baseline to Week 16. Secondary endpoints included changes in fasting blood glucose (FBG), postprandial blood glucose (PPBG), and safety outcomes.

**Results:**

Of 2,705 screened, 2,704 participants with T2DM were included in the analysis. Mean HbA1c decreased significantly from 8.78% (SD 1.16) at baseline to 7.38% (SD 0.98) at Week 16, with a mean reduction of −1.40% (SD 0.99; p < 0.0001). Significant reductions were also observed in FBG (−46.1 mg/dL) and PPBG (−72.0 mg/dL) from baseline to Week 16 (both p < 0.0001). Improvements in HbA1c levels were consistent across age and gender subgroups. No adverse events (AEs) were reported among participants with the available safety data.

**Conclusion:**

These findings demonstrate that imeglimin 1000 mg twice daily was associated with clinically meaningful improvements in glycemic control over 16 weeks and was generally well tolerated, supporting its effectiveness and safety for the management of T2DM in real-world settings.

## 1 Introduction

India accounts for a major global diabetes burden, with over 89.8 million adults affected by T2DM, a number expected to surpass 156.7 million by 2050 (1). Type 2 Diabetes Mellitus (T2DM) is a chronic metabolic disorder characterized by insulin resistance and progressive β-cell dysfunction, resulting in persistent hyperglycemia (2). This sustained hyperglycemia is associated with increased risk of long-term complications and mortality (3, 4). Early disease onset, rapid progression, and frequent comorbidities highlight the importance of safe and effective pharmacologic interventions to maintain glycemic control and reduce complications (5). Contributing factors include rapid urbanization, sedentary lifestyles, increased consumption of energy-dense diets, and genetic predisposition (6, 7).

Oral antidiabetic agents (OADs) remain the backbone of pharmacologic therapy for T2DM. These agents target distinct pathophysiologic abnormalities, including insulin resistance, impaired insulin secretion, and excessive hepatic glucose production (8). Major classes of OADs include biguanides, sulfonylureas, thiazolidinediones, α-glucosidase inhibitors, dipeptidyl peptidase-4 (DPP-4) inhibitors, sodium-glucose cotransporter-2 (SGLT2) inhibitors, and *glimins* (8).

While current OADs offer several therapeutic advantages, they often do not address all three primary defects of diabetes, which are excessive hepatic glucose production, impaired peripheral glucose uptake, and insufficient insulin secretion. Furthermore, most agents lower glucose predominantly through a single mechanism of action. Consequently, despite the availability of multiple OADs, achieving durable glycemic control in patients with T2DM remains challenging (8). Also, many widely used therapies are associated with limitations such as the risk of hypoglycemia, weight gain, gastrointestinal adverse effects, and declining efficacy over time (9, 10). Together, these limitations highlight the need for therapeutic options that provide sustained glycemic control, act through complementary mechanisms, and maintain a favorable safety and tolerability profile.

*Glimins* are a novel class of OADs, with imeglimin as the first-in-class agent (11). Unlike some traditional OADs, imeglimin offers a novel approach, leveraging both glucose-dependent insulinotropic and insulin-sensitizing properties through mitochondrial bioenergetics in key metabolic tissues, including pancreatic β-cells, liver, and skeletal muscles (11–13). These effects are achieved without increasing the risk of hypoglycemia or promoting weight gain, supporting a physiologically compatible approach to glycemic control (14–16). Imeglimin is currently the only *glimin* approved for clinical use, having received its first regulatory approval in Japan in 2021 (17) and subsequent approval in India for the treatment of adults with T2DM in 2022 (18, 19).

Randomized clinical studies, including TIMES 1, 2, and 3, have shown that imeglimin can effectively improve glycemic control while maintaining a favorable safety profile (20–22). These studies observed reductions in HbA1c levels with good overall tolerability. All of the TIMES phase 3 trials were conducted exclusively in the Japanese population, limiting the direct applicability of their findings to the Indian population.

To address this gap in the Indian setting, the INDI-TIMES, a multicenter retrospective study, was conducted across 191 sites and enrolled 8301 participants to provide real-world evidence supporting the effectiveness and tolerability of imeglimin in the Indian population. However, more real-world studies are required, as participants in clinical practice are far more heterogeneous with respect to baseline glycemic control, background therapies, comorbidity burden, renal function, and treatment persistence, all of which may influence observed effectiveness and safety outcomes.

In this context, the present study was conducted to evaluate the effectiveness and safety of imeglimin in the Indian population with T2DM treated in routine clinical practice. The primary objective was to assess the change in HbA1c from baseline to Week 16, while secondary objectives included evaluation of changes in FBG and PPBG, as well as assessment of safety and tolerability.

## 2 Methods

This prospective, non-interventional, single-arm, open-label, multi-center, observational study evaluated the effectiveness and safety of imeglimin in adults with T2DM. Eligible participants were enrolled during clinical visits at the discretion of the treating physician. The study concluded 16 weeks after the enrollment of the last participant, with an additional flexible 1-week timeframe for the safety visit. Study assessments were conducted under routine clinical conditions and comprised 3 visits: an initial (baseline) visit, an optional follow-up visit at Week 8, and an end-of-observation (final) visit at Week 16.

The study was conducted across 145 centers in India between July 2024 and April 2025. Participants were followed for 16 weeks after initiation of imeglimin. The study was conducted in accordance with the ethical principles of the Declaration of Helsinki and applicable Indian regulatory guidelines. The protocol was reviewed and approved by the Independent Ethics Committee (IEC) at each participating site, and written informed consent was obtained from all participants prior to any study-related activities.

### 2.1 Study population

Eligible participants included adults (≥18 years of age) of either gender with a confirmed diagnosis of T2DM who were prescribed imeglimin, either as monotherapy or in combination with other glucose-lowering therapies, independent of their decision to participate in the study. Participants were excluded if they had a diagnosis of T1DM, maturity-onset diabetes of the young, latent autoimmune diabetes in adults, gestational diabetes, or any hyperglycemic condition other than T2DM. Additional exclusion criteria included known pregnancy or breastfeeding (or planned pregnancy during the study period), participation in an interventional clinical study within 3 months, use of investigational drugs within 30 days, known contraindications to imeglimin, or any clinically significant condition that could interfere with study participation (**Supplementary Table 1**).

### 2.2 Study outcomes and assessments

Imeglimin was administered orally at a dose of one tablet (1000 mg) twice daily, in the morning and evening, with dose adjustments according to clinical judgment based on effectiveness and tolerability. Treatment adherence was assessed based on physician judgment and patient self-report.

Study assessments were performed at baseline, an optional interim visit at Week 8, and a final visit at Week 16. Participants who discontinued treatment prematurely were followed through the end of the observation period.

The primary endpoint was the mean change in HbA1c from baseline to Week 16. Secondary endpoints included change from baseline in FBG and PPBG at Week 16, and the occurrence of AEs and serious adverse events (SAEs) from post-baseline visits through Week 16. Laboratory parameters, vital signs, and physical examination findings were recorded at baseline and at Week 16. All laboratory assessments were performed in accordance with the standard operating procedures of each participating site.

### 2.3 Statistical analysis

All analysis were performed in the Overall Analysis Set, defined as participants who received at least one dose of imeglimin. Statistical analysis were descriptive and exploratory as the study was not designed to test predefined hypotheses. No formal sample size calculation was performed, consistent with standard practice for non-interventional observational studies.

All variables were analyzed descriptively using appropriate statistical methods: categorical variables were summarized using frequency tables (absolute and relative frequencies), and continuous variables were presented as participants (n), mean, standard deviation (SD), median, quartiles, minimum, and maximum. Categorical variables were summarized as counts (n) and percentages (%). Participants who received at least one dose of imeglimin were included in the analysis. Analyses were conducted using only available data, with no imputation for missing efficacy and safety data. As treatment decisions were made by physicians and data were collected under routine clinical practice conditions, the study may be subject to selection bias, variability in routine data recording, and confounding due to concomitant therapies and patient characteristics.

The primary endpoint was the change in HbA1c from baseline to Week 16. Actual values at baseline and Week 16, as well as changes from baseline, were summarized descriptively using the Overall Analysis Set. The secondary endpoint analysis included the change from baseline in FBG and PPBG at the end of Week 16, and the occurrence of AEs/SAEs related to tolerability from the post-baseline visit to the end of Week 16. Actual FBG and PPBG values at the baseline visit and Week 16 were summarized as descriptive statistics using the Overall Analysis Set. The significance of the change from baseline to Week 16 within the imeglimin treatment group was assessed using a paired *t*-test at the 5% level of significance.

Subgroup analysis of the primary outcome were performed according to age (18–45 years, 46–60 years, and >60 years) and sex. A paired *t*-test was performed at a 5% level of significance for each subgroup.

The incidence of AEs was summarized as frequencies and %, with corresponding 95% confidence intervals calculated using the Clopper–Pearson exact method. Changes in laboratory parameters and vital signs from baseline to Week 16 were summarized descriptively. Categorical shifts in vital signs and physical examination findings (normal, abnormal, not clinically significant, and clinically significant abnormal) were summarized by visit.

## 3 Results

A total of 2,705 participants were screened for the study and met the eligibility criteria. Treatment details for one participant were missing, resulting in an analysis population of 2,704 participants. Consequently, 2,704 participants constituted the Overall Analysis Set, which was used for demographic, baseline, and efficacy analysis.

The mean age of the participants was 54.8 years (SD 9.9). Overall, 77.3% (n = 2090) of participants were male, and 22.7% (n = 614) were female. The mean body weight was 71.17 kg (SD 10.9), and the mean height was 163.7 cm (SD 10.4). A total of 90.16% (n=2438) of participants in the Overall Analysis Set had a prior diagnosis of T2DM.

T2DM duration was available for 2,241 participants (82.9%) and was missing for 463 participants (17.1%); analysis were based on available data without imputation. Among participants with available data, the mean (SD) duration of T2DM was 6.1 years (SD 3.8), with a median of 5.5 years (**Table 1**).

**Table 1:** Summary of demographics and baseline characteristics.

Characteristic	Imeglimin 1000 mg (N = 2704)
Age (years)	
n	2704
Mean (SD)	54.8 (9.87)
Median	55.0
Q1, Q3	48.0, 61.0
Gender, n (%)	
Male	2090 (77.3)
Female	614 (22.7)
Height (cm)	
n	2675
Mean (SD)	163.7 (10.40)
Median	164.0
Q1, Q3	157.0, 170.0
Weight (kg)	
n	2675
Mean (SD)	71.17 (10.92)
Median	71.00
Q1, Q3	63.00, 79.00
T2DM History recorded	
Yes	2438 (90.16)
No	148 (5.47)
Missing	119 (4.40)
T2DM Duration (Years)	
n	2241
Mean (SD)	6.1 (3.88)
Median	5.5
Q1, Q3	3.41, 8.00

T2DM Duration, Years + (Months/12).

T2DM, Type 2 Diabetes Mellitus; SD, standard deviation.

Most participants previously received antidiabetic therapy, predominantly metformin (71.89%; n=1944) (**Figure 1**). Other anti-diabetic treatment included DPP-4 inhibitors such as sitagliptin (30.40%; n=822), followed by linagliptin (2.14%; n=58) and teneligliptin (1.96%; n=53). Sulfonylureas were also commonly prescribed, predominantly glimepiride (24.82%; n=671), along with gliclazide (5.21%; n=141) and glibenclamide (2.18%; n=59). SGLT-2 inhibitors were used in a notable proportion of participants, including dapagliflozin (11.87%; n=321) and empagliflozin (1.04%; n=28). Insulin use, including human insulin, insulin analogs, and combination regimens (e.g., insulin glargine, lispro, degludec, and premixed formulations), was observed in 1.5% participants (n=38), based on aggregation of all insulin-related entries. Other OADs, particularly voglibose, were prescribed in 7.77% (n=210) of participants, while thiazolidinediones, such as pioglitazone, accounted for 1.59% (n=43). Less frequently used agents included acarbose (0.59%; n=16), repaglinide (0.11%; n=3), and lobeglitazone (0.26%; n=7). A wide variety of fixed-dose OAD combinations (e.g., metformin with sulfonylureas, DPP-4 inhibitors, or SGLT-2 inhibitors) and other concomitant medications (e.g., antihypertensives, statins) were reported (**Supplementary Table 2**). Background treatment remained largely stable following initiation of imeglimin therapy, with additional concomitant antidiabetic medications prescribed in 0.6% (n=17) of participants at Week 8 and Week 16, respectively 1 (**Supplementary Table 3**).

**Figure 1:**
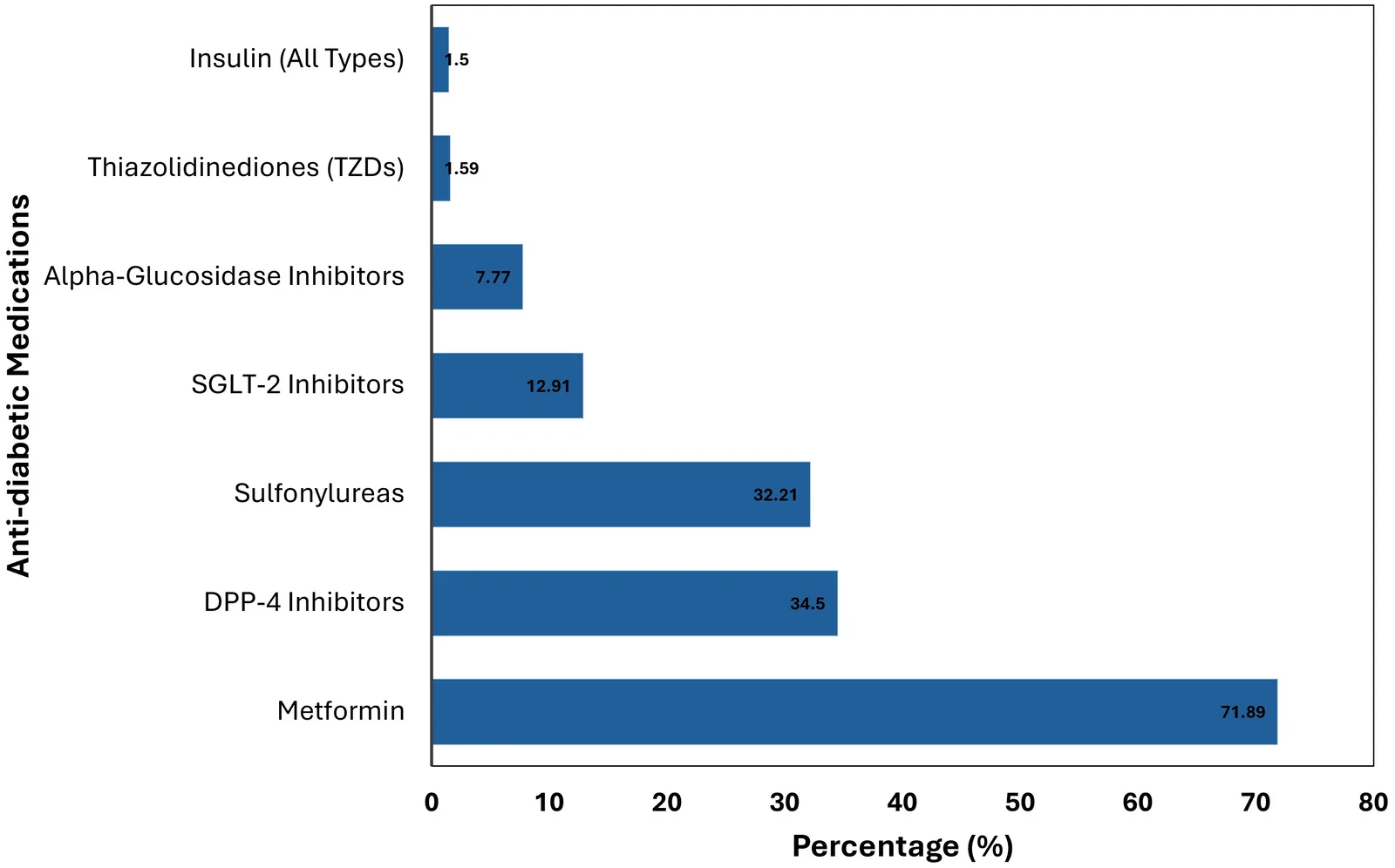
Distribution of the most commonly drugs used before imeglimin initiation. Percentages shown on the bars represent the proportion of participants receiving each medication class. SGLT-2, Sodium-Glucose Cotransporter-2; DPP-4, Dipeptidyl Peptidase-4.

### 3.1 Study treatment

At baseline, all 2704 enrolled participants followed the assigned treatment regimen. All participants (n=2704) received the 1000 mg dose of imeglimin. At Week 16, all participants (n=2704) followed the prescribed treatment regimen and were reported to be on the imeglimin 1000 mg twice daily regimen (**Supplementary Table 3**).

### 3.2 Efficacy results

The primary and secondary efficacy analysis for this post-marketing real-world evidence study were conducted using the Overall Analysis Set, comprising all participants who received at least one dose of imeglimin 1000 mg.

Mean HbA1c decreased from 8.78% (SD 1.16) at baseline (n=2,704) to 7.38% (SD 0.98) at Week 16 (n=2,704). The mean change in HbA1c from baseline to Week 16 was −1.40% (SD 0.99; 95% CI: −1.44 to −1.36; p < 0.0001), with a median change of −1.16% (Q1–Q3: −2.00 to −0.80). This indicates a statistically significant reduction in HbA1c from baseline (**Table 2**).

**Table 2:** Change in HbA1c (%) from baseline at the end of week 16 (overall analysis set).

Visit statistics	Imeglimin 1000 mg (N = 2704)	
	Observed	Change from baseline
Baseline		
n	2704	
Mean (SD)	8.78 (1.16)	
Median	8.60	
min, max	4.4, 14.3	
Q1, Q3	7.90, 9.30	
Week 16		
n	2704	2704
Mean (SD)	7.38 (0.98)	-1.40 (0.99)
Median	7.10	-1.16
min, max	3.5, 12.4	-5.8, 3.0
Q1, Q3	6.90, 7.90	-2.00, -0.80
95% CI for Mean		-1.44, -1.36
P-value		<0.0001

P-value derived from paired t-test (two-sided significance level α = 0.05).

Assessments reported as Week 16 were performed within the predefined visit window of Week 16.

HbA1c, glycated hemoglobin; SD, standard deviation.

In the Overall Analysis Set (n=2,704), FBG values were available for 2,702 participants at baseline, with 2 participants having missing data. At Week 16, data were available for 2,701 participants, with 3 participants having missing values. Mean FBG decreased from 182.66 mg/dL (SD 50.75) at baseline (n = 2,702) to 136.59 mg/dL (SD 35.53) at Week 16 (n = 2,701) (**Table 3**). The mean change in FBG from baseline to Week 16 was −46.06 mg/dL (95% CI: −47.64 to −44.47; p < 0.0001). The reduction in FBG from baseline to Week 16 was statistically significant.

**Table 3:** Change from baseline in FBG (mg/dL) at the end of week 16 (overall analysis set).

Visit statistics	Imeglyn^®^ 500/1000 (N = 2704)	
	Observed	Change from baseline
Baseline		
n	2702	
Missing	2	
Mean (SD)	182.66 (50.745)	
Median	175.00	
min, max	70, 390	
Q1, Q3	148.00, 204.00	
Week 16		
n	2701	2701
Missing	3	3
Mean (SD)	136.592 (35.5297)	-46.055 (41.9975)
Median	132.000	-38.000
min, max	0.79, 380.00	-249.00, 139.00
Q1, Q3	110.000, 160.000	-68.000, -20.000
95% CI for Mean		-47.64, -44.47
P-value		<.0001

Change from baseline = (Observed value – baseline value).

The mean for the “Change from Baseline” column is calculated based on participants with non-missing values at both the Baseline and Post-Baseline visits.

P-values were calculated using a paired t-test.

In the Overall Analysis Set (n=2,704), PPBG values were available for 2,702 participants at baseline, with 2 participants having missing data. At Week 16, PPBG data were available for 2,701 participants, with 3 participants having missing values. Mean PPBG decreased from 257.38 mg/dL (SD 78.46) at baseline (n=2702) to 185.35 mg/dL (SD 41.85) at Week 16 (n = 2,701). The mean change in PPBG from baseline to Week 16 was −72.01 mg/dL (95% CI: −74.79 to −69.22; p < 0.0001) (**Table 4**). The reduction in PPBG from baseline to Week 16 was statistically significant (**Figures 2**, **3**).

**Table 4:** Change from baseline in PPBG (mg/dL) at the end of week 16 (overall analysis set).

Visit statistics	Imeglyn^®^ 500/1000 (N = 2704)	
	Observed	Change from baseline
Baseline		
n	2702	
Missing	2	
Mean (SD)	257.38 (78.462)	
Median	253.00	
min, max	1, 2701	
Q1, Q3	211.00, 295.00	
Week 16		
n	2701	2701
Missing	3	3
Mean (SD)	185.35 (41.854)	-72.01 (73.797)
Median	183.00	-60.00
min, max	0, 390	-2491, 236
Q1, Q3	155.00, 210.00	-105.00, -33.00
95% CI for Mean		-74.79, -69.22
P-value		<.0001

Change from baseline = (Observed value – baseline value).

The mean for the “Change from Baseline” column is calculated based on participants with non-missing values at both the Baseline and Post-Baseline visits.

P-values were calculated using a paired t-test.

**Figure 2:**
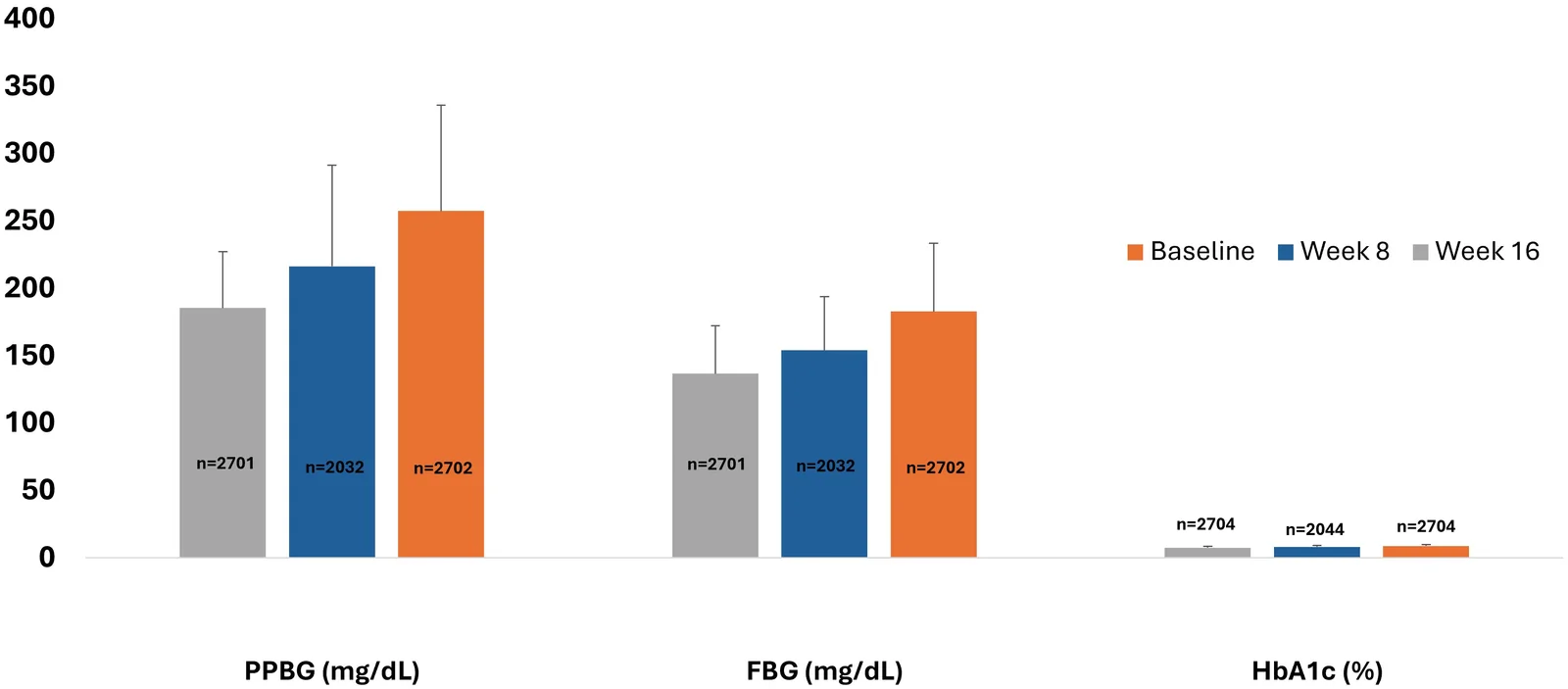
Effect of imeglimin on glycemic parameters over 16 (± 1) weeks. Error bars represent standard deviation. Assessments reported as Week 8 and Week 16 were performed within the predefined visit window of Week 8 and Week 16, respectively HbA1c, Glycated Hemoglobin; FBG, Fasting Blood Glucose; PPBG, Postprandial Blood Glucose

**Figure 3:**
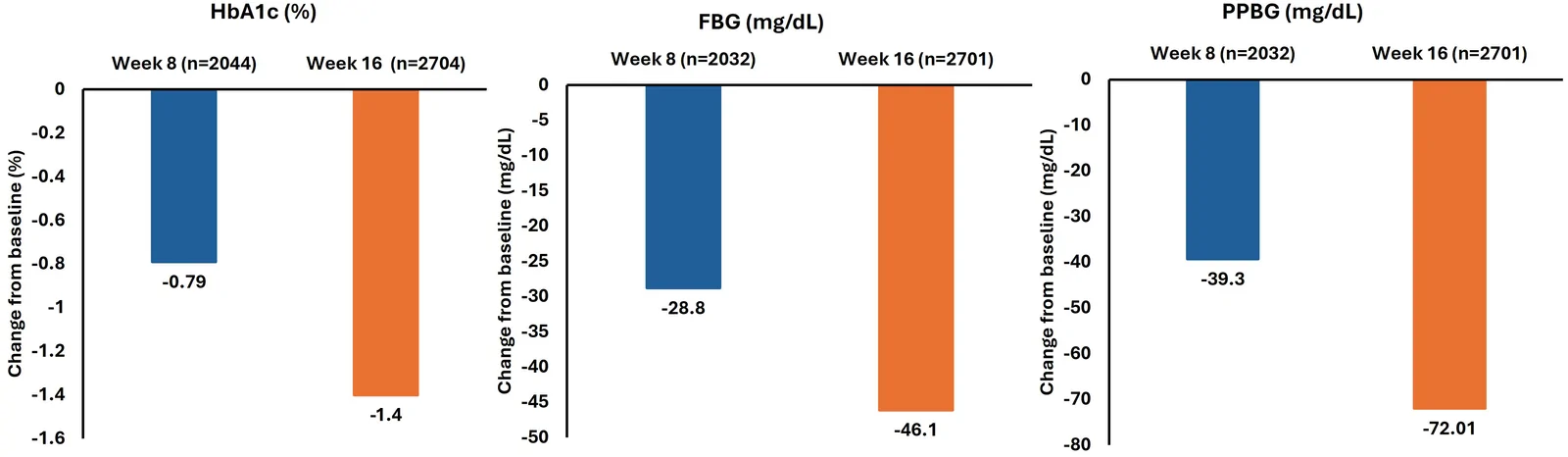
Change from baseline in HbA1c, FBG, and PPBG at week 8 and week 16 (± 1) during treatment with imeglimin. Mean change from baseline values was calculated using data from participants with non-missing values at both baseline and the corresponding post-baseline visit. Assessments reported as Week 8 and Week 16 were performed within the predefined visit window of Week 8 and Week 16, respectively HbA1c, Glycated Hemoglobin; FBG, Fasting Blood Glucose; PPBG, Postprandial Blood Glucose.

### 3.3 Subgroup analysis

At Week 16, treatment with imeglimin 1000 mg twice daily was associated with statistically significant reductions in HbA1c across all age subgroups. Among participants aged 18–45 years (n=459), mean HbA1c decreased from 8.45% (SD 0.99) at baseline to 7.29% (SD 0.85) at Week 16. The mean change from baseline was −1.16% (95% CI: −1.24 to −1.08; p < 0.0001. In participants aged 46–60 years (n=1475), mean HbA1c decreased from 8.76% (SD 1.12) at baseline to 7.33% (SD 0.92) at Week 16, corresponding to a mean change from baseline of −1.43% (95% CI: −1.48 to −1.38; p < 0.0001). Among participants >60 years (n=770), mean HbA1c decreased from 9.01% (SD 1.27) at baseline to 7.53% (SD 1.14) at Week 16, with a mean change from baseline of −1.48% (95% CI: −1.55 to −1.41; p < 0.0001). A significant reduction in HbA1c from baseline to Week 16 was observed across all age groups (**Supplementary Table 4**).

Among male participants (n=2,090), the mean HbA1c declined from 8.78% (SD 1.15) at baseline to 7.35% (SD 0.94) at Week 16 corresponding to a mean change from baseline of −1.44% (95% CI: −1.48 to −1.39; p < 0.0001)while in female participants (n=614), mean HbA1c decreased from 8.76% (SD 1.19) at baseline to 7.49% (SD 1.09) at Week 16, corresponding to a mean change from baseline of −1.27% (95% CI: −1.35 to −1.20; p < 0.0001) (**Figures 4**, **5**).

**Figure 4:**
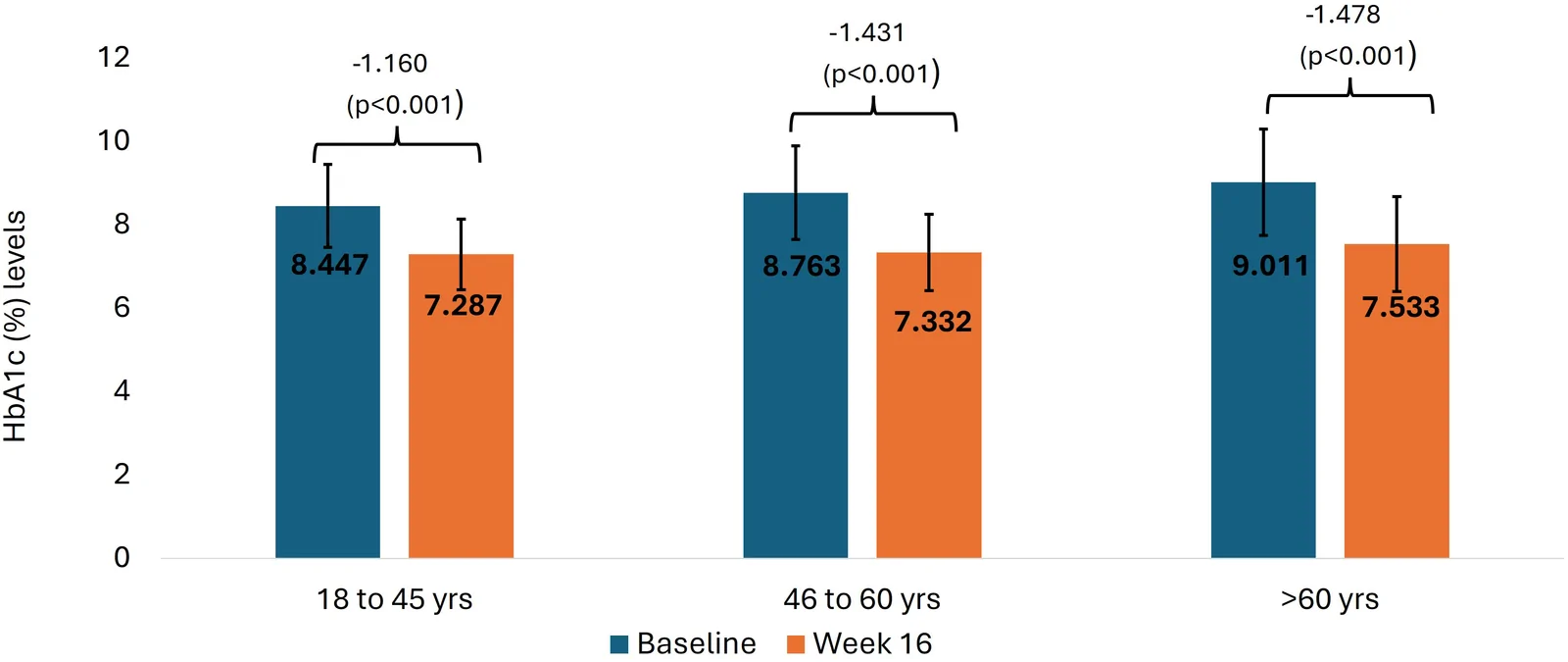
Change in HbA1c levels by age from baseline to 16 (± 1) weeks. Error bars represent standard deviation. Assessments reported as Week 16 were performed within the predefined visit window of Week 16. HbA1c, Glycated Hemoglobin; yrs, years.

**Figure 5:**
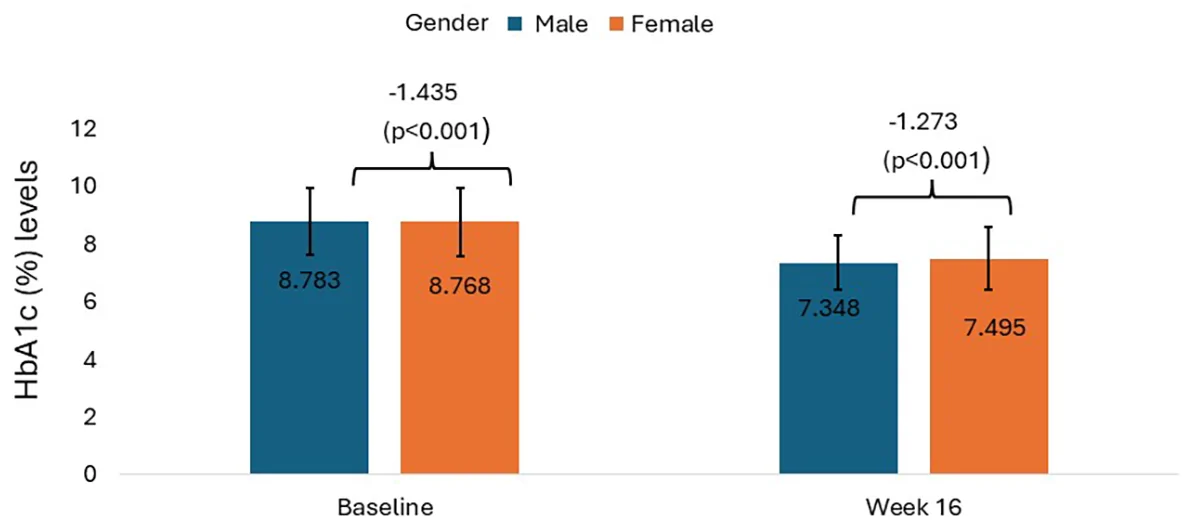
Change in HbA1c levels by gender from baseline to 16 (± 1) weeks. Error bars represent standard deviation. Assessments reported as Week 16 were performed within the predefined visit window of Week 16. HbA1c, Glycated Hemoglobin.

### 3.4 Safety results

Among participants with available safety assessments, no AEs were documented at Week 8 or Week 16. At the optional Week 8 visit, safety data were available for 1,093 participants (40.4%), whereas AE information was missing for 1,611 participants (59.6%). At Week 16, safety data were available for 2,434 participants (90.0%) and were missing for 270 participants (10.0%). Therefore, the absence of documented AEs pertains only to participants with available safety assessments and should not be interpreted as confirming the absence of AEs in the entire study population (**Supplementary Table 5**).

Changes in laboratory and clinical parameters were evaluated over the 16-week treatment period. Renal function, as assessed by serum creatinine, remained largely stable over time. Mean creatinine was 1.33 mg/dL at baseline (n=1,651) and showed a transient increase at Week 8 to 1.72 mg/dL at Week 8 (n=879), and decreased to 1.20 mg/dL at Week 16 (n=1,567).

Mean Total cholesterol decreased from 197.0 mg/dL at baseline (n=776) to 185.1 mg/dL at Week 16 (n=557), corresponding to a mean change from baseline of -21.8 mg/dL. Mean High-Density Lipoprotein (HDL) increased from 47.0 mg/dL at baseline (n=759) to 51.2 mg/dL at Week 16 (n=488) with a mean change of +2.0 mg/dL. Mean Very Low-Density Lipoprotein (VLDL) levels remained largely stable over the study period, with baseline (n=739) values of 69.6 mg/dL and 83.4 mg/dL at Week 16 (n=466), corresponding to a mean change from baseline of +0.3 mg/dL. Triglyceride levels decreased over the study period. Mean triglycerides reduced from 154.6 mg/dL at baseline (n=764) to 135.3 mg/dL at Week 16 (n=586), with a mean change from baseline of −25.9 mg/dL (**Figure 6**). At baseline, all vital signs and physical examination findings were within normal limits. Post-baseline data for vital signs and physical examination were missing for participants at Weeks 8 and 16.

**Figure 6:**
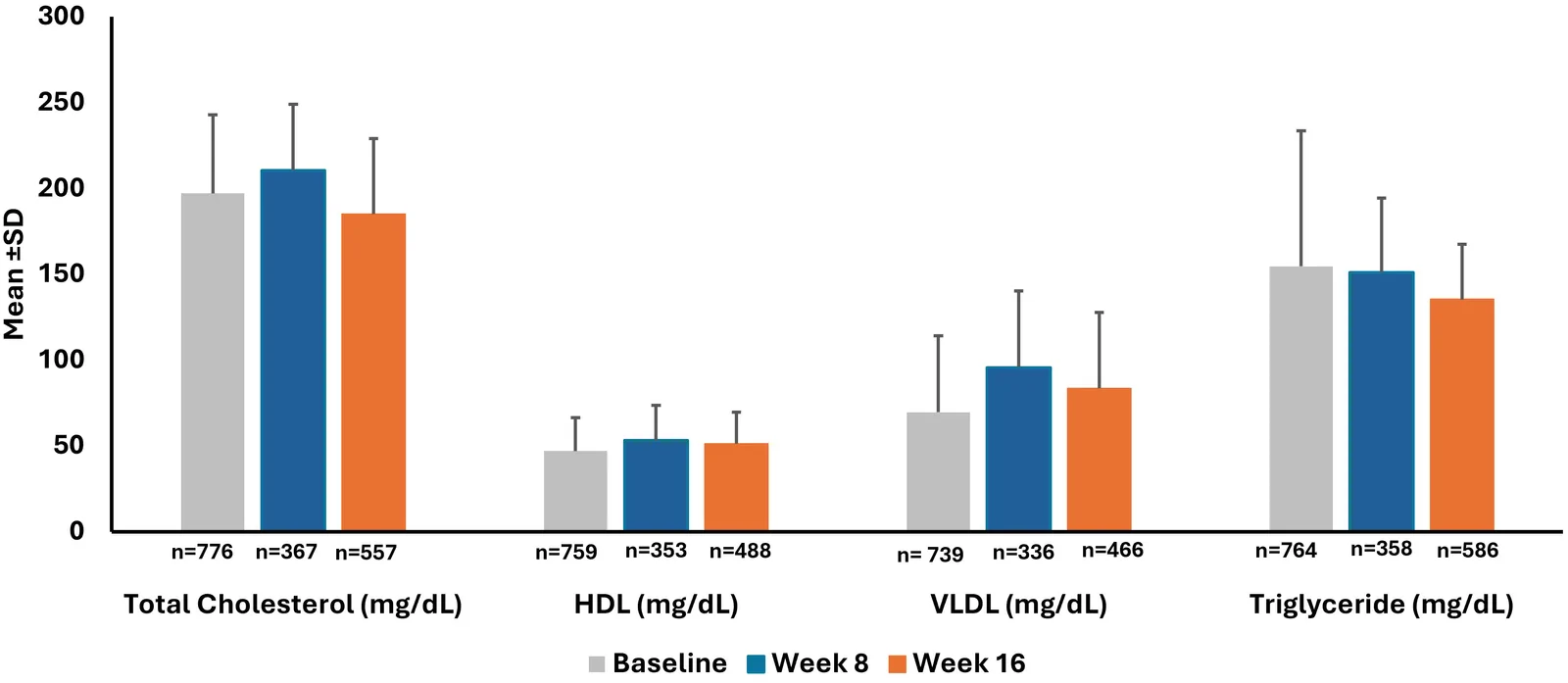
Effect of imeglimin on lipid profile parameters over 16 (± 1) weeks. Error bars represent standard deviation. Assessments reported as Week 8 and Week 16 were performed within the predefined visit window of Week 8 and Week 16, respectively. HDL, High-Density Lipoprotein; VLDL, Very Low-Density Lipoprotein.

## 4 Discussion

This prospective, non-interventional, single-arm, open-label, multi-center observational study provides real-world evidence on the effectiveness and safety of imeglimin 1000 mg twice daily in 2,704 adults with T2DM treated under routine clinical practice across 145 centers in India. At Week 16, treatment with imeglimin was associated with statistically significant and clinically meaningful improvements in glycemic control, as evidenced by reductions in HbA1c, FBG, and PPBG levels. The treatment was well tolerated, with no AEs reported among participants with available safety data, and no new safety signals were identified.

A mean reduction in HbA1c of 1.40% (SD 0.99) from baseline to Week 16 was observed in the overall analysis population, representing a clinically meaningful improvement. The participants had a baseline HbA1c of 8.78%, reflecting a real-world population with a prior diagnosis of T2DM (90.16%) in whom existing treatment regimens had failed to achieve adequate glycemic control. The observed improvement in HbA1c, therefore, suggests improved glycemic control following initiation of imeglimin. This magnitude of HbA1c reduction is consistent with findings from previously published randomized controlled trials and real-world studies of imeglimin (20–23).

The findings of the present study are consistent with those reported in the INDI-TIMES study conducted in Indian adults with T2DM (24). In the INDI-TIMES study, treatment with imeglimin was associated with clinically meaningful reductions in HbA1c (1.12%; p < 0.0001), accompanied by parallel improvements in FBG (-29.41 mg/dL) and PPBG (-62.41 mg/dL), indicating a consistent improvement in overall glycemic control over the 3-month follow-up period. Similarly, the PRISM study, a prospective real-world study conducted in a metformin-intolerant Indian population, demonstrated a mean HbA1c reduction of -1.26% at 6 months, with even greater reductions (up to -1.86%) observed when imeglimin was used as part of combination therapy (25). Despite differences in study duration, patient characteristics, and clinical settings, these studies demonstrate that imeglimin was associated with improved glycemic control in the Indian population across diverse clinical settings.

These findings are similar to those observed in previously conducted randomized controlled trials, including the TIMES phase 3 clinical trials conducted in Japan. TIMES 1 demonstrated a placebo-adjusted HbA1c reduction of -0.87% at 24 weeks with imeglimin monotherapy (p < 0.001) (20). TIMES 2 reported HbA1c reductions of 0.46% with monotherapy, 0.56–0.92% with oral combination therapies, and 0.12% with injectable GLP-1 receptor agonist combination therapy over 52 weeks, with the greatest reduction (0.92%) observed in combination with DPP-4 inhibitors (21). The TIMES 3 trial demonstrated an adjusted mean HbA1c reduction of 0.60% at Week 16 versus placebo, which was sustained through Week 52, with a mean reduction of 0.64% from baseline (22). Consistent with these findings, a retrospective longitudinal study conducted in Japan demonstrated reductions in HbA1c of 0.7% at 3 months, 1.1% at 6 months, and 1.0% at 12 months following the initiation of imeglimin (23). Previous studies demonstrated that each 1% reduction in HbA1c is associated with a reduced risk of complications associated with T2DM, highlighting the clinical relevance of the glycemic improvement observed in these studies (4, 26).

In addition to HbA1c, significant reductions in both FBG (-46.05 mg/dL) and PPBG (-72.01 mg/dL) were observed at Week 16. The improvement in PPBG was particularly pronounced, suggesting effective control of postprandial hyperglycemia, which is a key contributor to overall glycemic burden in participants with T2DM. These findings are consistent with those of the INDI-TIMES study, which similarly reported significant reductions in FBG (−29.41 mg/dL) and PPBG (−62.41 mg/dL) at 3 months in clinical practice.

Subgroup analysis demonstrated that reductions in HbA1c were consistent across all age groups, including elderly participants. Although older participants had higher baseline HbA1c levels, the reduction was comparable and numerically greater than that observed in younger participants. These findings are broadly consistent with prior randomized controlled trials and real-world data, which have demonstrated glycemic improvement across demographic subgroups (20–22, 24). With respect to gender, reductions were observed in both male (n=2,090) and female participants (n=614); however, the substantially smaller number of female participants limits the generalizability of these findings.

The current study additionally provides important insights into the role of imeglimin in Indian adults with T2DM. Compared with Western populations, Indians with T2DM exhibit a distinct metabolic phenotype characterized by higher insulin resistance, increased visceral adiposity, early β-cell dysfunction, and pronounced postprandial hyperglycemia despite relatively lower BMI (27–29). These pathophysiological abnormalities often contribute to rapid disease progression and difficulty achieving durable glycemic control. Imeglimin, through its action on insulin sensitivity and glucose-dependent insulin secretion mediated by improvement in mitochondrial function and cellular bioenergetics, may therefore offer therapeutic advantages in this population (11, 13, 23, 30). The improvements observed in HbA1c, FBG, and PPBG in the present study may be consistent with the potential of imeglimin to target multiple underlying metabolic defects rather than acting through a single glucose-lowering mechanism. Similar findings have also been reported in previous Indian and Asian real-world studies evaluating imeglimin therapy (23, 24). This multi-targeted effect may contribute to more stable glycemic control, which is increasingly recognized as an important therapeutic goal in T2DM management (31).

Most participants were receiving background antidiabetic therapy, predominantly metformin, along with other commonly used oral agents. Background treatment remained largely stable throughout the study period, with minimal modifications to concomitant antidiabetic medications occurring in 5.9% of participants at baseline and 0.6% at Week 16. This relative stability may minimize potential confounding due to changes in background therapy and support the interpretation that the observed improvements in glycemic parameters are associated with imeglimin therapy.

No AEs were reported at week 16 and week 8, although 90% of data was available at week 16, with limited data, i.e., 40.4% at week 8 (optional visit). Additionally, a meta-analysis of randomized controlled trials has confirmed the favorable safety and tolerability profile of imeglimin across diverse treatment settings (15).

A favorable but modest improvement in lipid profile was observed over 16 weeks in the present study, with decreases in total cholesterol (−21.8 mg/dL) and triglycerides (−25.9 mg/dL), along with a slight rise in HDL (+2.0 mg/dL) and VLDL (+0.3 mg/dL). Comparable trends with greater reductions were reported in the INDI-TIMES study for total cholesterol (−56.34 mg/dL), triglycerides (−74.61 mg/dL), and HDL (+4.68 mg/dL). These reductions may be linked to imeglimin’s proposed action on mitochondrial function and reduction of oxidative stress, which can influence lipid metabolism (13). A modest reduction in serum creatinine levels was also observed at Week 16 (−0.13 mg/dL). However, the interpretation of these findings should consider the potential confounding effects of concomitant lipid-lowering therapies and other antidiabetic medications that may also affect these parameters. In this study, the interpretation of changes in vital signs and physical examination findings was limited because post-baseline data at Weeks 8 and 16 were missing for most participants.

This study has several strengths. The large sample size (N = 2,704) with participants treated in routine clinical practice across 145 centers ensures a diverse clinical population. This enhances the heterogeneity of the study population and supports generalizability to the Indian T2DM population in real-world settings. The prospective design reduces bias and enables assessment of glycemic changes at pre-specified time points, while the non-interventional approach allows outcomes to reflect routine clinical practice.

The findings of this study should be interpreted in light of several limitations. Although background antidiabetic treatment remained largely stable during the study period, the potential influence of concomitant therapies and other confounding factors cannot be excluded. Therefore, the observed improvements in glycemic parameters should be interpreted as being associated with imeglimin treatment and cannot be attributed exclusively to imeglimin.T2DM duration was unavailable for 17.1% of participants. As this was a descriptive baseline characteristic and missing values were not imputed, the reported duration of T2DM may not fully represent the overall study population.

This study included both male and female patients, although a predominance of male patients in the study population may limit the robustness and generalizability of the gender-specific findings. Therefore, these subgroup results should be interpreted as descriptive and should not be considered definitive evidence of differences in treatment response between males and females. A limitation of the study is that safety data were unavailable for 59.6% of participants at Week 8 and 10.0% at Week 16, which limits definitive interpretation of the absence of documented AEs in the overall study population. In addition, the follow-up duration was limited to 16 weeks, and longer-term glycemic control, durability of response, and long-term safety outcomes could not be assessed. Despite these limitations, the cohort reflects real-world conditions, providing practical insights into the effectiveness, safety, and tolerability of imeglimin in routine clinical practice.

This prospective, non-interventional, multicenter observational study conducted in India showed that treatment with imeglimin 1000 mg twice daily was associated with statistically significant improvements in HbA1c, FBG, and PPBG over 16 weeks among adults with T2DM in the real-world setting. No AEs were documented among participants with available safety assessments during the observation period. Further, long-term studies, particularly in treatment-naive populations and those including active comparators, may help provide additional evidence on the long-term effectiveness and safety of imeglimin.

## Data Availability

The original contributions presented in the study are included in the article/**Supplementary Material**. Further inquiries can be directed to the corresponding author.
