# Supplementary material for: Effectiveness and safety of imeglimin in routine clinical practice among Indian patients with type 2 diabetes mellitus: a prospective, non-interventional, real-world observational study (INDI-TIMES 2.0)
Source: Front Endocrinol (Lausanne). 2026 Oct 12;17:1973270. doi: 10.3389/fendo.2026.1973270 (PMC13696222; doi:10.3389/fendo.2026.1973270)
Supplement: Supplementary file 1 [file Table1.docx]

Supplementary Material

## 1. Supplementary Tables

Supplementary Table 1. Inclusion and exclusion criteria

| **Inclusion criteria** | **Exclusion criteria** |
| --- | --- |
| - Informed consent obtained before any study-related activities. - Adult male and/or non-pregnant non-lactating female patients aged 18 years or above. - Patient diagnosed with T2DM. - The decision to initiate treatment with commercially available Imeglimin has been made by the treating physician before and independently from the patient’s decision to participate in this study. | - Diagnosed with type 1 diabetes mellitus, maturity-onset diabetes of the young, latent autoimmune diabetes in adults, gestational diabetes, or any other hyperglycemic state other than T2DM. - Women known to be pregnant or breastfeeding, or women planning to become pregnant during the conduct of the study. - Participation in another T2DM clinical study that involves any clinical intervention or administration of an investigational drug within 3 months prior to enrolment into the study. - Any contraindications for imeglimin, including hypersensitivity to the active substances or any of the excipients as specified in the imeglimin local label. - Treatment with any investigational drug within 30 days prior to enrolment into the study. - Subject with history or presence of clinically significant disease, which, as per the Investigator's discretion, might interfere with the subject's participation in the study. |

Supplementary Table 2. Concomitant Medications

| **Concomitant Medications** | **Imeglimin 1000 mg (N = 2704) n (%)** |
| --- | --- |
| A To Z | 1 (0.04) |
| Acarbose | 16 (0.59) |
| Amlodipine | 9 (0.33) |
| Amlodipine + Chlorthalidone | 1 (0.04) |
| Amlong | 2 (0.07) |
| Aspirin (Ecosprin) | 1 (0.04) |
| Atorvastatin | 3 (0.11) |
| Atorvastatin + Aspirin | 5 (0.18) |
| Basaglar | 3 (0.11) |
| Beugli | 1 (0.04) |
| Bisoprolol | 1 (0.04) |
| Bisoprolol + Telmisartan | 1 (0.04) |
| Calcilogix | 1 (0.04) |
| Calcium | 2 (0.07) |
| Chlorthalidone + Telmisartan | 1 (0.04) |
| Cholecalciferol | 1 (0.04) |
| Cilnidipine | 1 (0.04) |
| D-Rise | 1 (0.04) |
| Dapagliflozin | 321 (11.87) |
| Dapagliflozin + Linagliptin | 2 (0.07) |
| Dapagliflozin + Linagliptin + Metformin | 2 (0.07) |
| Dapagliflozin + Metformin | 26 (0.96) |
| Dapagliflozin + Metformin + Sitagliptin | 6 (0.22) |
| Dapagliflozin + Sitagliptin | 9 (0.33) |
| Dapanorm | 20 (0.74) |
| Darolutazar | 1 (0.04) |
| Dilnip | 2 (0.07) |
| Domperidone | 1 (0.04) |
| Dpp-4 Inhibitor | 30 (1.11) |
| Empagliflozin | 28 (1.04) |
| Empagliflozin + Linagliptin | 2 (0.07) |
| Enalapril | 3 (0.11) |
| Esomeprazole + Itopride | 4 (0.15) |
| Febuxostat | 1 (0.04) |
| Gamera | 1 (0.04) |
| Gemer | 4 (0.15) |
| Glador | 2 (0.07) |
| Glibenclamide | 59 (2.18) |
| Gliclazide | 141 (5.21) |
| Gliclazide + Metformin | 7 (0.26) |
| Gliclazide Xr | 1 (0.04) |
| Glimepiride | 671 (24.82) |
| Glimepiride + Dapagliflozin + Metformin | 3 (0.11) |
| Glimepiride + Metformin | 74 (2.74) |
| Glimepiride + Metformin + Pioglitazone | 2 (0.07) |
| Glimepiride + Metformin + Voglibose | 3 (0.11) |
| Glimepiride Xr | 1 (0.04) |
| Glimisave | 1 (0.04) |
| Glimisave M2 Forte | 1 (0.04) |
| Glimy | 1 (0.04) |
| Glipizide | 5 (0.18) |
| Glucobay | 1 (0.04) |
| Gluconorm | 3 (0.11) |
| Glycomet | 2 (0.07) |
| Glycomet Gp 0.5 | 1 (0.04) |
| Hcqs | 1 (0.04) |
| Human Insulin | 2 (0.07) |
| Human Insulin 100 Iu | 2 (0.07) |
| Insulin | 16 (0.59) |
| Insulin (Mixtard 30/70) | 1 (0.04) |
| Insulin 30/70 | 1 (0.04) |
| Insulin Degludec | 2 (0.07) |
| Insulin Degludec + Insulin Aspart | 3 (0.11) |
| Insulin Glargine | 3 (0.11) |
| Insulin Humalog | 1 (0.04) |
| Insulin Insugen | 1 (0.04) |
| Insulin Lispro | 1 (0.04) |
| Insulin Novomix | 1 (0.04) |
| Insulin Wosulin | 4 (0.15) |
| Jalra | 2 (0.07) |
| Levothyroxine | 3 (0.11) |
| Linagliptin | 58 (2.14) |
| Lobeglitazone | 7 (0.26) |
| Lobotror | 1 (0.04) |
| Loyzide-Xr | 1 (0.04) |
| Lupislin | 1 (0.04) |
| Metformin | 1944 (71.89) |
| Metformin Xr | 1 (0.04) |
| Neurobion | 1 (0.04) |
| Olmesartan + Amlodipine | 1 (0.04) |
| Omeprazole | 1 (0.04) |
| Pioglitazone | 43 (1.59) |
| Piroxicam | 1 (0.04) |
| Pregabalin | 1 (0.04) |
| Rabeprazole | 1 (0.04) |
| Ramipril | 1 (0.04) |
| Razor | 1 (0.04) |
| Reclide | 1 (0.04) |
| Remogliflozin | 3 (0.11) |
| Remogliflozin + Vildagliptin | 3 (0.11) |
| Repaglinide | 3 (0.11) |
| Repaglinide + Voglibose | 1 (0.04) |
| Rosumac | 2 (0.07) |
| Rosuvastatin | 16 (0.59) |
| Rosuvastatin + Aspirin | 4 (0.15) |
| Rosuvastatin + Clopidogrel | 2 (0.07) |
| Rozat | 1 (0.04) |
| Saroglitazar | 7 (0.26) |
| Semaglutide | 2 (0.07) |
| Sglt2 Inhibitors | 3 (0.11) |
| Sitagliptin | 822 (30.40) |
| Sitagliptin + Metformin | 7 (0.26) |
| Sitagliptin + Pioglitazone + Metformin | 1 (0.04) |
| Sitared | 1 (0.04) |
| Tamsulosin + Finasteride | 1 (0.04) |
| Telmisartan | 13 (0.48) |
| Telmisartan + Amlodipine | 1 (0.04) |
| Telmisartan + Cilnidipine | 1 (0.04) |
| Teneligliptin | 53 (1.96) |
| Thyroxine | 2 (0.07) |
| Timolol | 2 (0.07) |
| Torsemide | 4 (0.15) |
| Triglynase | 2 (0.07) |
| Ursodeoxycholic Acid | 1 (0.04) |
| Valsartan + Salbutamol | 1 (0.04) |
| Vildagliptin + Dapagliflozin | 4 (0.15) |
| Vildagliptin + Dapagliflozin + Metformin | 5 (0.18) |
| Vildagliptin + Metformin | 7 (0.26) |
| Vildagliptin Sr | 2 (0.07) |
| Voglibose | 210 (7.77) |
| Voglistar | 2 (0.07) |
| Xilingo | 1 (0.04) |
| Zomelis | 7 (0.26) |
| Zucapride | 2 (0.07) |

Supplementary Table 3. Treatment Regime and Anti-Diabetic Medications (Overall Analysis Set)

| **Visit** | **Parameter** | **Imeglimin 1000 mg (N = 2704) n (%)** |
| --- | --- | --- |
| Baseline | Participants followed the treatment regime |  |
|  | Yes | 2704 (100) |
|  | No | 0 |
|  | Participants are on which IP regime |  |
|  | 500 mg | 0 |
|  | 1000 mg | 2704 (100) |
|  | Have there been any changes in diabetic medications? |  |
|  | Yes | 160 (5.9) |
|  | No | 2365 (87.5) |
|  | Missing | 179 (6.6) |
|  | If yes, Drug name |  |
|  | Missing | 101 (3.7) |
|  | Acarbose | 1 (0.0) |
|  | Dapagliflozin | 4 (0.2) |
|  | Empagliflozin | 1 (0.0) |
|  | Gliclazide | 1 (0.0) |
|  | Glimeperide | 9 (0.3) |
|  | Glimiperide | 2 (0.1) |
|  | Imeglimin | 9 (0.3) |
|  | Imeglyn | 15 (0.6) |
|  | Metformin | 11 (0.4) |
|  | Sitagliptin | 5 (0.2) |
|  | Vildagliptin | 1 (0.0) |
|  | | |
| Week 8 | Participants followed the treatment regime |  |
|  | Yes | 1080 (39.9) |
|  | No | 0 |
|  | Missing | 1624 (60.1) |
|  | Participants are on which IP regime |  |
|  | 500 mg | 0 |
|  | 1000 mg | 1080 (39.9) |
|  | Missing | 1624 (60.1) |
|  | Have there been any changes in diabetic medications? |  |
|  | Yes | 17 (0.6) |
|  | No | 1061 (39.2) |
|  | Missing | 1626 (60.1) |
|  | If yes, Drug name |  |
|  | Missing | 7 (0.3) |
|  | Dapagliflozin | 1 (0.0) |
|  | Imeglimin | 2 (0.1) |
|  | Metformin | 6 (0.2) |
|  | Sitagliptin | 1 (0.0) |
|  | | |
| Week 16 | Participants followed the treatment regime |  |
|  | Yes | 2704 (100) |
|  | No | 0 |
|  | Participants are on which IP regime |  |
|  | 500 mg | 0 |
|  | 1000 mg | 2704 (100) |
|  | Have there been any changes in diabetic medications? |  |
|  | Yes | 17 (0.6) |
|  | No | 1215 (44.9) |
|  | Missing | 1472 (54.4) |
|  | If yes, Drug name |  |
|  | Missing | 8 (0.3) |
|  | Glyclazide | 1 (0.0) |
|  | Imeglimin | 2 (0.1) |
|  | Metformin | 1 (0.0) |
|  | Sitagliptin | 2 (0.1) |
|  | Vildagliptin | 3 (0.1) |

*Assessments reported as Week 8 and Week 16 were performed within the predefined visit window of Week 8 and Week 16, respectively

Supplementary Table 4. Subgroup analysis of change in HbA1c (%) from baseline at the end of week 16 (Overall Analysis Set)

|  | **Imeglyn® 500/1000 (N = 2704)** | | |
| --- | --- | --- | --- |
|  |  | | |
| **Visit  Statistics** | **Observed** | **Change from baseline** | |
|  | | | |
| **Age** |  |  | |
| **18 to 45** |  |  | |
| **Baseline** |  |  | |
| n | 459 |  | |
| Mean (SD) | 8.447 (0.9945) |  | |
| Median | 8.200 |  | |
| min, max | 5.70, 14.30 |  | |
| Q1, Q3 | 7.800, 9.100 |  | |
| **Week 16** |  |  | |
| n | 459 | 459 | |
| Mean (SD) | 7.287 (0.8452) | -1.160 (0.8630) | |
| Median | 7.100 | -1.100 | |
| min, max | 5.00, 12.00 | -5.30, 2.30 | |
| Q1, Q3 | 6.800, 7.900 | -1.600, -0.700 | |
| 95% CI for Mean |  | -1.24, -1.08 | |
| P-value |  | <.0001 | |
| **46 to 60** |  |  |  |
| **Baseline** |  |  |  |
| n | 1475 |  |  |
| Mean (SD) | 8.763 (1.1207) |  |  |
| Median | 8.600 |  |  |
| min, max | 4.40, 13.50 |  |  |
| Q1, Q3 | 7.900, 9.400 |  |  |
| **Week 16** |  |  |  |
| n | 1475 | 1475 |  |
| Mean (SD) | 7.332 (0.9181) | -1.431 (1.0087) |  |
| Median | 7.100 | -1.200 |  |
| min, max | 3.50, 12.00 | -4.70, 2.40 |  |
| Q1, Q3 | 6.900, 7.800 | -2.000, -0.800 |  |
| 95% CI for Mean |  | -1.48, -1.38 |  |
| P-value |  | <.0001 |  |

| **>60** |  |  | | |
| --- | --- | --- | --- | --- |
| **Baseline** |  |  | | |
| n | 770 |  | | |
| Mean (SD) | 9.011 (1.2739) |  | | |
| Median | 8.900 |  | | |
| min, max | 5.10, 13.10 |  | | |
| Q1, Q3 | 8.100, 9.800 |  | | |
| **Week 16** |  |  | | |
| n | 770 | 770 | | |
| Mean (SD) | 7.533 (1.1356) | -1.478 (1.0092) | | |
| Median | 7.200 | -1.300 | | |
| min, max | 5.40, 12.40 | -5.80, 3.00 | | |
| Q1, Q3 | 6.900, 7.900 | -2.100, -0.800 | | |
| 95% CI for Mean |  | -1.55, -1.41 | | |
| P-value |  | <.0001 | | |
| **Sex** |  | |  |  |
| **Male** |  | |  | |
| **Baseline** |  | |  | |
| n | 2090 | |  | |
| Mean (SD) | 8.783 (1.1521) | |  | |
| Median | 8.600 | |  | |
| min, max | 4.40, 14.30 | |  | |
| Q1, Q3 | 7.900, 9.400 | |  | |
| **Week 16** |  | |  | |
| n | 2090 | | 2090 | |
| Mean (SD) | 7.348 (0.9378) | | -1.435 (0.9993) | |
| Median | 7.100 | | -1.200 | |
| min, max | 3.50, 12.40 | | -5.30, 3.00 | |
| Q1, Q3 | 6.800, 7.900 | | -2.000, -0.800 | |
| 95% CI for Mean |  | | -1.48, -1.39 | |
| P-value |  | | <.0001 | |
| **Female** |  | |  | |
| **Baseline** |  | |  | |
| n | 614 | |  | |
| Mean (SD) | 8.768 (1.1924) | |  | |
| Median | 8.660 | |  | |
| min, max | 5.90, 13.40 | |  | |
| Q1, Q3 | 7.900, 9.300 | |  | |
| **Week 16** |  | |  | |
| n | 614 | | 614 | |
| Mean (SD) | 7.495 (1.0983) | | -1.273 (0.9543) | |
| Median | 7.200 | | -1.000 | |
| min, max | 5.00, 12.00 | | -5.80, 2.50 | |
| Q1, Q3 | 6.900, 7.900 | | -1.900, -0.700 | |
| 95% CI for Mean |  | | -1.35, -1.20 | |
| P-value |  | | <.0001 | |
| Change from baseline = (Observed value – baseline value). | | | |  |
| The mean for the “Change from Baseline” column is calculated based on participants with non-missing values at both the Baseline and Post-Baseline visits. | | | |  |
| Note 1: P-values are calculated by using paired t-test. | | | |  |

Supplementary Table 5. Summary of Adverse Events Occurrence by Visit (Overall Analysis Set)

| **Visit** | **Adverse Event** | **Imeglimin 1000 mg (N = 2704) n (%)** | **95% CI** |
| --- | --- | --- | --- |
| Week 8 | Participants experienced any adverse event |  |  |
|  | Yes | 0 | (NE, NE) |
|  | No | 1093 (40.4) | (38.56, 42.30) |
|  | Missing | 1611 (59.6) | (57.70, 61.44) |
|  | | | |
| Week 16 | Participants experienced any adverse event |  |  |
|  | Yes | 0 | (NE, NE) |
|  | No | 2434 (90.0) | (88.82, 91.12) |
|  | Missing | 270 (10.0) | (8.88, 11.18) |
